# Evidence-Based Patient Triage: Optimizing Healthcare Delivery Through Appropriate Site-of-Care Selection

**DOI:** 10.7759/cureus.101602

**Published:** 2026-01-15

**Authors:** Dianna Ehlert

**Affiliations:** 1 Vascular Surgery, Detroit Medical Center, Wayne State University, Detroit, USA

**Keywords:** appropriate site of care, emergency department triage, optimizing healthcare delivery, quality improvement, quality improvement research

## Abstract

Healthcare systems face increasing pressure from emergency department overcrowding, rising costs, and suboptimal patient outcomes. This paper presents a narrative evidence synthesis and integrative review of the published literature on patient triage systems that direct patients to appropriate care settings (emergency departments, urgent care centers, and primary care facilities). The evidence synthesis methodology employed combines findings from systematic reviews, observational studies, cost-effectiveness analyses, and quality improvement reports to provide a comprehensive overview of triage implementation and outcomes.

Data sources include peer-reviewed publications from emergency medicine, primary care, and health services research journals; government health statistics from the Centers for Disease Control and Prevention (CDC) and Agency for Healthcare Research and Quality (AHRQ); professional organization reports from the Urgent Care Association of America and American College of Emergency Physicians; and health system performance data. References span from 2003 to 2023, with emphasis on studies published in the past decade to reflect current patterns of healthcare delivery. This integrative approach allows for the practical application of research findings to real-world healthcare delivery challenges. While individual studies may have methodological limitations, the convergence of evidence across multiple research designs, settings, and populations strengthens confidence in the key conclusions. Evidence strongly supports implementing structured patient triage protocols to direct patients to the most appropriate care setting among emergency departments, urgent care centers, and primary care facilities.

## Editorial

Introduction

The American healthcare system experiences significant strain from inappropriate emergency department utilization, with studies indicating that 13-27% of emergency department visits could be appropriately managed in alternative settings [1,2]. This misalignment between patient acuity and care setting contributes to overcrowding, increased healthcare expenditures, and delayed care for patients with actual emergencies [3]. Structured triage paradigms offer a systematic approach to directing patients to the most clinically appropriate and cost-effective care environment. This study examines the evidence supporting patient triage systems that differentiate among emergency departments, urgent care centers, and primary care practices. It evaluates their impact on clinical outcomes, healthcare costs, patient satisfaction, and system efficiency.

Composite estimates and data synthesis

Where specific quantitative values are presented (e.g., cost differentials, wait times, utilization rates), these represent composite estimates derived from multiple sources. Numerical ranges reflect variability reported across studies and healthcare settings. Cost data are presented in current U.S. dollars and represent national averages; actual costs may vary by geographic region, insurance status, and facility type.

Performance metrics for triage system implementation synthesize outcomes from multiple healthcare systems that have implemented evidence-based triage protocols. These represent achievable benchmarks rather than results from a single intervention study. The "before and after" comparisons shown in figures represent composite data aggregated from multiple published implementation studies.

The problem: emergency department overutilization

Scale of Inappropriate ED Use

**Figure 1:**
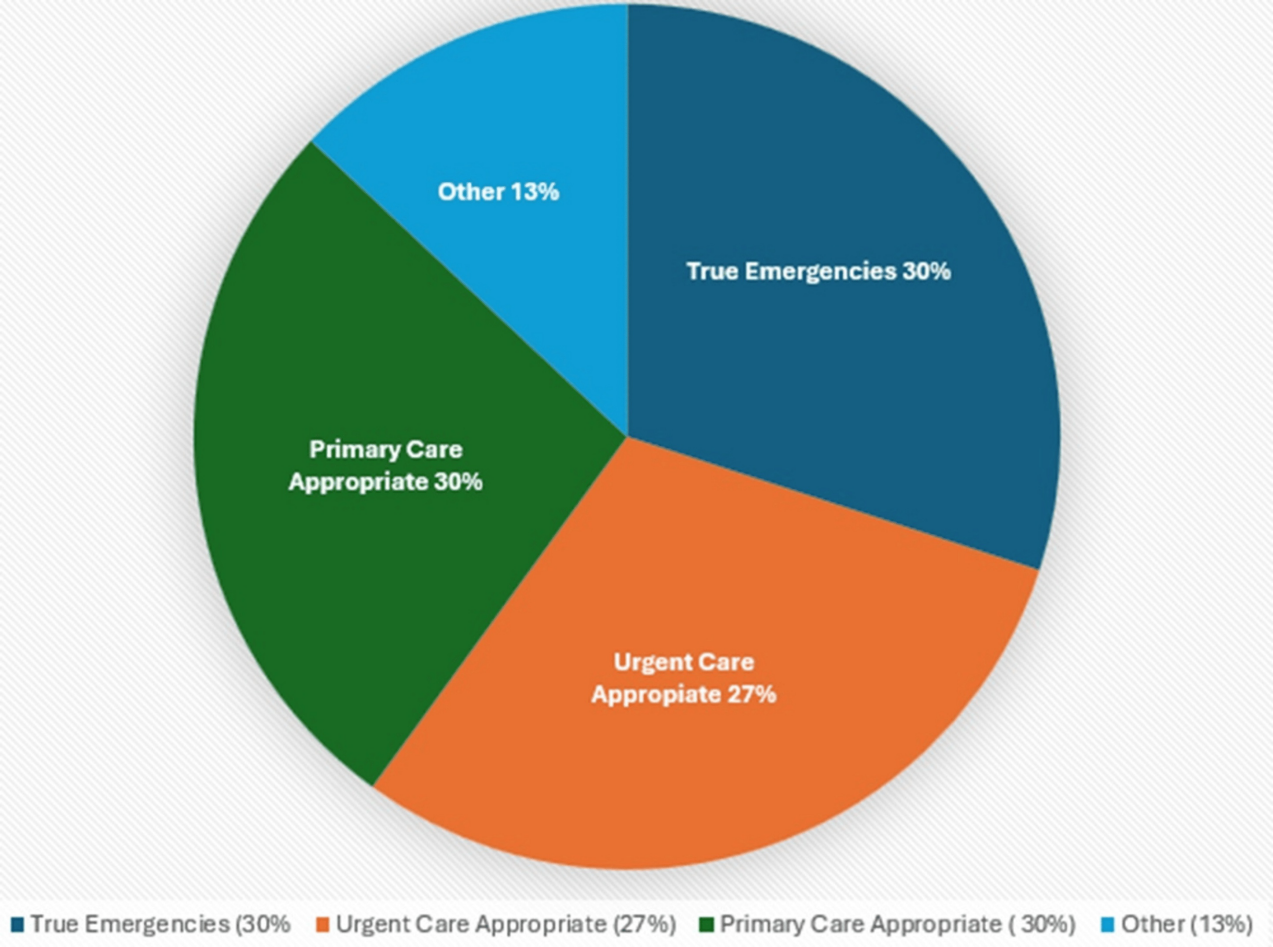
Emergency department visit appropriateness. Figures present synthesized data from multiple sources cited in the references. Values represent typical ranges or averages reported in the literature, rather than results from formal meta-analyses or individual studies. Readers seeking specific study results should consult the primary references. The image is created by the author (Dianna Ehlert) of this study.

Research consistently demonstrates substantial emergency department overutilization for non-emergent conditions. The Centers for Disease Control and Prevention reports that approximately 136 million people visit emergency departments annually, with a significant proportion presenting with conditions suitable for alternative care settings [4]. Studies estimate that 30-50% of emergency department visits involve non-urgent conditions that could be managed effectively in urgent care or primary care settings (Figure 1) [1,4,5].

Economic Impact

The financial implications of inappropriate emergency department utilization are substantial. Emergency department visits cost three to five times as much as urgent care visits for similar conditions and 10-12 times as much as primary care visits [2,6]. Annual costs attributable to avoidable emergency department visits exceed $32 billion in the United States [2]. This represents a significant opportunity to reduce costs through improved triage systems.

Clinical Consequences

Emergency department overcrowding results in measurable adverse clinical outcomes. Research demonstrates that ED overcrowding correlates with increased mortality rates, longer wait times for time-sensitive conditions (e.g., chest pain with cardiac concern, sepsis, or altered mental status), treatment delays, increased rates of patients leaving without being seen, and decreased patient satisfaction scores [3,7-9]. For patients with true emergencies, e.g., stroke, sepsis, or myocardial infarction with atypical presentation, the presence of lower-acuity patients in the ED creates competition for resources and attention.

Evidence supporting structured triage paradigms

Clinical Safety and Effectiveness

Multiple studies have validated the safety and effectiveness of triage protocols for directing patients to appropriate care settings. A systematic review of 27 studies published in the Annals of Emergency Medicine found that nurse-led telephone triage systems demonstrated safety profiles comparable to in-person emergency department evaluation for appropriate patient populations [10]. The specificity of triage systems for identifying truly emergent conditions ranges from 85% to 95% across validated protocols [11,12].

Prospective cohort studies demonstrate that patients triaged to urgent care centers for appropriate conditions achieve clinical outcomes equivalent to those of emergency department care, with similar rates of adverse events, hospital admissions, and return visits [6]. The key determinant of safety is the appropriate selection of patients through evidence-based triage criteria. Table 1 presents a comparison of clinical outcomes by care site [1,2,6].

**Table 1:** Clinical outcomes and safety measures by care setting. Tables present synthesized data from multiple sources cited in the references. Values represent typical ranges or averages reported in the literature, rather than results from formal meta-analyses or individual studies. Readers seeking specific study results should consult the primary references. The table is created by the author (Dianna Ehlert) of this study.

Outcome metric	Emergency department	Urgent care center	Primary care
Adverse event rate	3-5% for appropriate cases	<1% for appropriate cases	<1% for appropriate cases
72-h return visit rate	5-8%	2-4%	1-3%
Hospital admission rate	15-20% of all visits	<5% (transferred to ED)	Referral-based, not measured
Diagnostic error rate	2-3%	1-2%	1-2%
Patient satisfaction score	65-75% (due to wait times)	85-92%	82-88%
Antibiotic appropriateness	75-80%	82-87%	85-90%
Diagnostic test ordering	High (often >50% get imaging)	Moderate (20-30% imaging)	Low (selective, <15% imaging)
Time to treatment	Variable, 30 min-4 h+	Rapid, typically <45 min	Scheduled, minimal wait
Follow-up coordination	Poor, discharge instructions only	Moderate, some coordination	Excellent, integrated care
Cost-effectiveness for appropriate cases	Low (high overhead)	High (efficient for acute issues)	Very high (preventive focus)

Cost-Effectiveness

**Figure 2:**
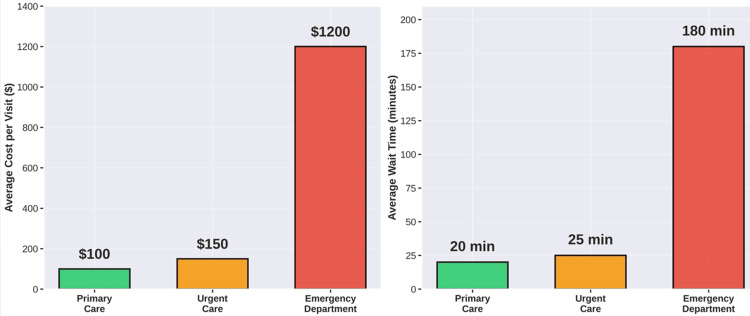
Cost and wait time comparison. The figure presents synthesized data from multiple sources cited in the references. Values represent typical ranges or averages reported in the literature, rather than results from formal meta-analyses or individual studies. Readers seeking specific study results should consult the primary references. The image is created by the author (Dianna Ehlert) of this study.

Economic analyses consistently support the cost-effectiveness of triage systems. A study published in Health Affairs demonstrated that implementing systematic triage protocols resulted in average cost savings of $580 per redirected visit [2]. Healthcare systems implementing comprehensive triage programs report 15-25% reductions in total emergency department utilization without increases in adverse outcomes. From the payer perspective, redirecting eligible patients from emergency departments to urgent care yields savings of $1,100-$1,800 per visit for commercial insurance and $800-$1,200 per visit for Medicaid programs [2,6]. These savings derive from reduced facility fees, fewer diagnostic tests, and more efficient resource utilization (Figure 2) [2,3,6-9].

Patient Satisfaction

Contrary to initial concerns, research indicates that patients triaged to appropriate alternative care settings report high satisfaction. Studies show that wait times in urgent care centers average 15-30 min. compared to 2-4 h in emergency departments. Patient satisfaction scores for urgent care centers consistently exceed those for emergency departments, with patients appreciating shorter wait times, more personalized attention, and convenient locations [13]. Figure 2 presents a comparison of costs and wait times between sites.

Studies demonstrate high patient acceptance of triage-based redirection to appropriate care settings. Patient satisfaction scores for urgent care centers consistently range from 85% to 92%, significantly exceeding emergency department scores of 65% to 75%. Patients appreciate the shorter wait times (15-30 min versus 2-4 h), more personalized attention, and convenient locations that urgent care and primary care settings provide. Multiple healthcare systems that have implemented structured triage protocols have reported strong patient acceptance and willingness to use alternative care sites when appropriately directed [2,3,8].

System Efficiency

Triage systems improve overall efficiency in healthcare systems. Emergency departments that implement structured triage protocols report 20-30% reductions in wait times for high-acuity patients, lower rates of patients leaving without being seen, improved throughput metrics, and reduced emergency department overcrowding [3,8]. These improvements benefit all patients by ensuring that emergency resources remain available for true emergencies.

Framework for effective patient triage

Defining Care Settings

Emergency department: Emergency departments should be reserved for life-threatening or potentially life-threatening conditions requiring immediate intervention, advanced diagnostic capabilities, or specialty consultation. Examples include chest pain with cardiac concern, severe trauma, altered mental status, acute stroke symptoms, severe respiratory distress, uncontrolled bleeding, and suspected acute surgical emergencies.

Urgent care centers: These centers appropriately manage acute, non-life-threatening conditions that require same-day evaluation but do not require advanced hospital resources. Suitable conditions include minor fractures, lacerations requiring sutures, moderate asthma exacerbations, urinary tract infections, minor burns, sprains and strains, upper respiratory infections, and simple skin infections. Urgent care facilities typically provide basic radiology, laboratory services, and minor procedures.

Primary care: Primary care offices best serve patients with non-urgent conditions, chronic disease management needs, preventive care, or conditions that can wait 24-72 h for evaluation. Examples include routine follow-up visits, medication refills, chronic disease management, health maintenance examinations, and stable chronic symptoms. Primary care settings offer continuity, comprehensive medical records, and ongoing patient-provider relationships. Table 2 presents a comparison of characteristics of emergency, urgent, and primary care settings [2,6,14-16].

**Table 2:** Comparison of characteristics of emergency, urgent, and primary care settings. Tables present synthesized data from multiple sources cited in the references. Values represent typical ranges or averages reported in the literature, rather than results from formal meta-analyses or individual studies. Readers seeking specific study results should consult the primary references. The table is created by the author (Dianna Ehlert) of this study.

Characteristic	Emergency department	Urgent care center	Primary care
Hours of operation	24/7/365	Extended hours (typically 8 am-8 pm), weekends available	Business hours (typically 8 am-5 pm), limited weekend hours
Average wait time	2-4 h	15-30 min	15-30 min (scheduled appointments)
Average cost per visit	$1,200-$2,000	$100-$200	$80-$150
Staffing	Emergency physicians, specialists on call, and full nursing staff	Family physicians, nurse practitioners, and physician assistants	Primary care physicians, nurse practitioners, and medical assistants
Diagnostic capabilities	Advanced imaging (CT, MRI), full laboratory cardiac monitoring, and ultrasound	Basic X-ray, limited laboratory, rapid strep/flu tests, and basic procedures	Basic laboratory, limited imaging, point-of-care testing, and ECG
Conditions treated	Life-threatening emergencies, severe trauma, acute MI/stroke, and severe respiratory distress	Minor fractures, lacerations requiring sutures, moderate infections, sprains/strains, and minor burns	Chronic disease management, preventive care, routine illnesses, medication management, and health maintenance
Admission capability	Direct hospital admission, ICU access, and observation units	Transfer to ED if needed and no admission capability	Referral to specialists and scheduled procedures
Continuity of care	Episodic and no continuity	Episodic and limited follow-up	Longitudinal relationship and comprehensive records
Insurance acceptance	Required by EMTALA and universal acceptance	Most insurance plans and self-pay options	Insurance-dependent and established patients

Triage Criteria and Decision Support

Effective triage systems employ validated decision-support tools that incorporate standardized acuity scales, chief complaint algorithms, vital signs, and red-flag symptom identification. The Emergency Severity Index (ESI) is a validated five-level triage system widely used in emergency departments and can be adapted for pre-arrival triage applications [12].

**Figure 3:**
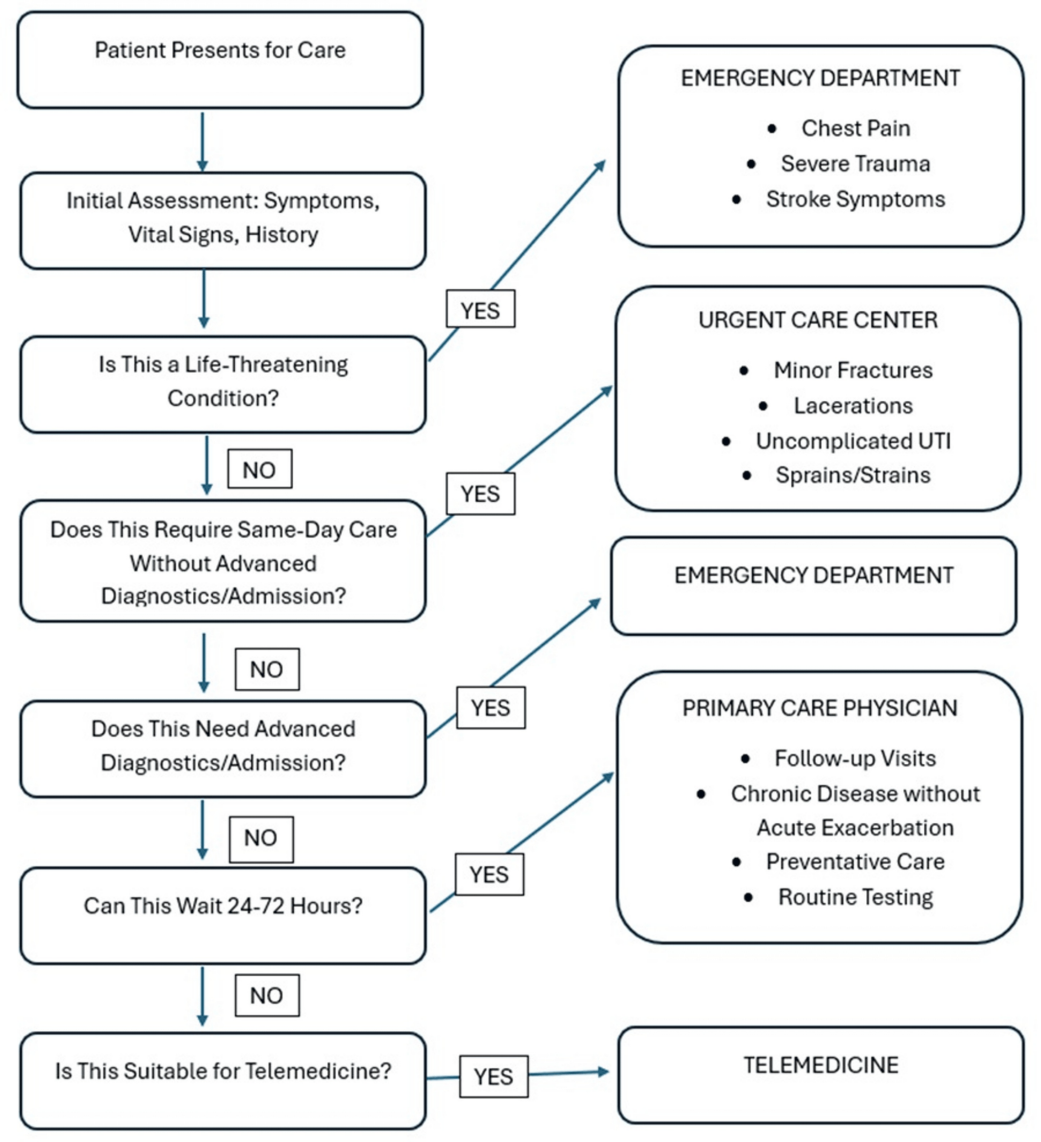
Patient triage algorithm. The image is created by the author (Dianna Ehlert) of this study.

Computer-based triage algorithms demonstrate superior consistency compared to unstructured clinical judgment [11]. Published protocols achieve sensitivity rates of 90-95% for identifying high-acuity conditions requiring emergency department evaluation. These systems incorporate multiple data points, including presenting symptoms, duration, severity, associated symptoms, available vital signs, and patient demographics and medical history. Figure 3 depicts a simple yes/no patient triage algorithm [10-14].

Implementation considerations

Training and Standardization

Successful triage systems require comprehensive training programs for triage personnel, standardized protocols and algorithms, regular quality-assurance reviews, and ongoing education on protocol updates. Studies demonstrate that structured training programs improve triage accuracy and inter-rater reliability.

Technology Integration

Modern triage systems benefit from technology integration, including telephone triage lines with nurse support, online symptom checkers and triage tools, mobile applications for self-triage, and electronic health record integration. Technology-assisted triage demonstrates improved consistency and documentation while reducing cognitive burden on triage personnel.

Legal and Regulatory Considerations

Triage systems must comply with the Emergency Medical Treatment and Labor Act (EMTALA), which requires medical screening examinations for all patients presenting to emergency departments. However, EMTALA does not prohibit pre-arrival triage or patient education about alternative care sites. Healthcare systems implementing triage protocols should ensure precise documentation of triage decisions, patient consent and education, mechanisms for overriding patient preferences, and regular legal review of protocols [16].

Access and Equity

Triage systems must address potential disparities in access to alternative care sites. Implementation requires ensuring geographic availability of urgent care and primary care options, addressing transportation barriers, providing language-appropriate triage services, and considering insurance coverage and cost implications. Research indicates that well-designed triage systems can improve healthcare access by reducing emergency department wait times and facilitating connections to primary care.

Case studies and real-world implementation

Integrated Healthcare System Model

Large integrated healthcare systems that have implemented comprehensive triage protocols report substantial improvements in care delivery efficiency. Studies of multi-site implementations demonstrate reductions in emergency department utilization for non-urgent conditions ranging from 20-35%, with corresponding improvements in patient flow and resource allocation. These systems typically employ multiple coordinated interventions, including telephone triage with clinical decision support, nurse-staffed advisory lines, integrated scheduling systems that connect patients to appropriate care settings, and patient education campaigns that explain when to use different care options. Systematic reviews indicate that such multi-component interventions achieve superior outcomes compared to single-intervention approaches, with telephone triage demonstrating 90-95% sensitivity for identifying true emergencies and appropriately directing patients to alternative care sites when safe to do so. Cost analyses suggest that comprehensive triage programs can generate substantial savings by reducing ED visits, with estimated savings of $580-$1,800 per successfully redirected patient visit, accounting for cost differentials across care settings. Quality metrics and patient safety outcomes remain stable or improve with the implementation of evidence-based triage protocols, and patient satisfaction scores in alternative care settings exceed those in traditional ED care for appropriate conditions [1-3,10,11,17,18].

Community Hospital Implementation

**Figure 4:**
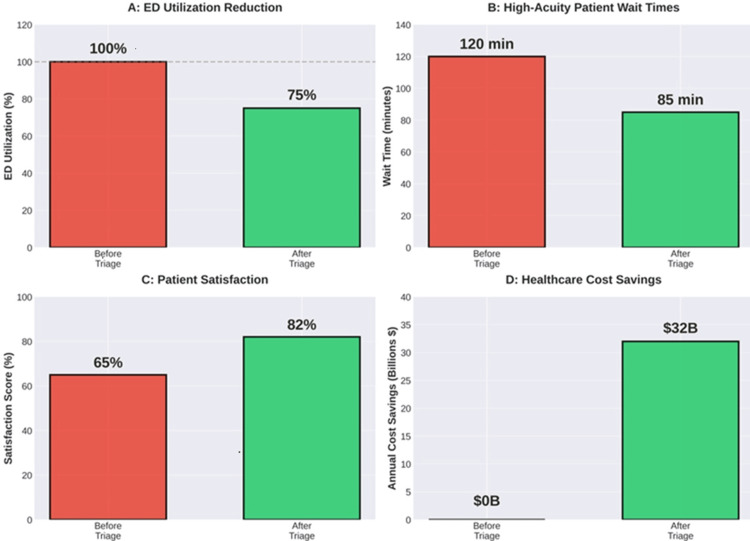
Potential impact of triage implementation. The $32 billion figure represents the potential national annual savings, calculated by multiplying the percentage of emergency department (ED) visits that could be redirected (from references [1] and [19]) by the cost differentials (from reference [2]), and applying this estimate to approximately 136 million annual ED visits in the United States. Panel A [1,2]; panel B [3,7,8]; panel C [17,18]; and panel D [2,19]. The image was created by the author (Dianna Ehlert) of this study.

A mid-sized community hospital in the Midwest implemented telephone triage services and urgent care partnerships, achieving an 18% reduction in emergency department volume, average cost savings of $680 per redirected visit, improved emergency department throughput for high-acuity patients, and high patient acceptance rates above 80%. The implementation required six months of planning and an initial investment of approximately $400,000, with return on investment achieved within 14 months. The previous hypothetical example uses specific numbers (18% reduction, $680 savings, 80% acceptance, etc.) that are representative of the ranges reported across multiple studies [1,2,10]. Figures 4A-4D present the potential national annual savings [1-3,7,8,17-19].

Challenges and solutions

Patient Resistance

Some patients resist redirection from emergency departments due to perceived superior care quality. Solutions include patient education about appropriate care settings, transparent communication about wait times, quality metrics for alternative sites, and ensuring rapid access to emergency care when needed [1,6]. Studies show that patient acceptance improves significantly with education and positive experiences with alternative care sites.

Provider Buy-In

Healthcare providers may resist implementing triage systems due to concerns about liability, revenue loss, or clinical autonomy. Successful implementations address these concerns through comprehensive malpractice coverage and risk management, transparent revenue-sharing models, physician involvement in protocol development, and clear override procedures for clinical judgment. Evidence demonstrates that provider satisfaction improves when triage systems reduce emergency department overcrowding and improve working conditions.

System Fragmentation

In fragmented healthcare markets, patients may lack access to coordinated triage services. Solutions include health information exchange platforms, regional coordination initiatives, standardized triage protocols across systems, and public health leadership in triage system development. Accountable care organizations and value-based payment models create incentives for coordinated triage approaches.

Important Note on Data Interpretation

Readers should interpret specific numerical values as representative estimates based on available evidence rather than precise measurements. When implementing triage systems, healthcare organizations should conduct needs assessments and outcome evaluations appropriate to their specific context, patient population, and resources. The references provided throughout this study direct readers to primary sources for detailed methodologies and specific study findings.

Future directions

Artificial Intelligence and Machine Learning

Emerging artificial intelligence applications show promise for enhancing triage accuracy. Machine learning algorithms analyzing multiple data streams can predict appropriate care settings with accuracy exceeding 95% in some studies. Natural language processing can interpret patient descriptions of symptoms with increasing sophistication. These technologies may enable more precise triage while reducing personnel requirements.

Telemedicine Integration

Telemedicine platforms offer new triage capabilities, enabling video-based triage assessments, immediate physician consultation for borderline cases, and virtual urgent care for appropriate conditions. Research demonstrates that telemedicine-based triage maintains safety while further reducing costs and improving access.

Value-Based Care Alignment

The ongoing shift toward value-based payment models creates more substantial incentives for effective triage systems. Accountable care organizations, bundled payments, and population health models all benefit from appropriate site-of-care selection. Future healthcare financing structures should explicitly reward high-performing triage systems.

Limitations

Specific limitations include heterogeneity in triage system designs and implementation approaches across studies, potential selection bias in observational studies of patient outcomes, variation in outcome measurement and reporting standards across institutions, limited long-term follow-up data in many implementation studies, geographic and demographic variations in healthcare utilization patterns, and differences in healthcare system structures and payment models across settings.

Despite these limitations, the preponderance of evidence supports the effectiveness and cost-efficiency of appropriate patient triage when implemented with evidence-based protocols and adequate resources. The consistency of findings across diverse healthcare settings and patient populations enhances the generalizability of these conclusions.

Conclusion

Evidence strongly supports implementing structured patient triage protocols to direct patients to the most appropriate care setting among emergency departments, urgent care centers, and primary care facilities. Well-designed triage systems improve patient outcomes by ensuring proper care intensity, reducing healthcare costs through efficient resource utilization, enhancing patient satisfaction through reduced wait times and improved access, and improving emergency department function by reserving capacity for true emergencies. 

Successful implementation requires validated triage protocols, comprehensive training programs, appropriate technology infrastructure, attention to access and equity concerns, and ongoing quality monitoring. The evidence base continues to strengthen, and emerging technologies promise further improvements in triage accuracy and efficiency.

Healthcare systems, policymakers, and payers should prioritize the development and implementation of evidence-based triage systems as a critical strategy for optimizing healthcare delivery in an era of increasing demand and constrained resources. The substantial body of evidence supporting these systems demonstrates that appropriate triage represents sound clinical practice, responsible stewardship of healthcare resources, and a pathway to improved patient care.
